# Percutaneous Endovascular Extraction of a Fractured Central Line Catheter From the Intracardiac Space Utilizing a Snare: A Case Report

**DOI:** 10.7759/cureus.98416

**Published:** 2025-12-03

**Authors:** Arindam Pande, Durlabh Debbarma, Ajanta Samanta, Saurav Das, Sourav Datta

**Affiliations:** 1 Cardiology, Manipal EM Bypass Hospital, Kolkata, IND; 2 Obstetrics and Gynaecology, Manipal EM Bypass Hospital, Kolkata, IND; 3 Oncosurgery, Manipal EM Bypass Hospital, Kolkata, IND

**Keywords:** central venous catheter, endovascular, fracture, intracardiac foreign bodies, peripherally inserted central catheters, retrieval, snare

## Abstract

Fracture and migration of a central venous catheter is an uncommon but serious complication. Fragments can embolize to the right atrium or ventricle, potentially causing life-threatening arrhythmias, pseudoaneurysms, perforations, or thromboembolism. We present the case of a 44-year-old woman with a fractured catheter from a prior insertion for buccal mucosa carcinoma. Fluoroscopy revealed the fragment in the right atrium, and it was successfully retrieved via a percutaneous endovascular approach using a snare, without immediate complications. This case highlights the efficacy and safety of endovascular retrieval for such critical events.

## Introduction

Central venous access devices are essential for sustained intravenous therapy in oncology, critical care, and long-term medical management. Despite their utility, they carry a risk of complications, with catheter fracture and migration being rare but demanding urgent intervention. These devices are particularly indicated for long-term therapies, such as chemotherapy, which can sclerose peripheral vessels [1,2]. Venous port catheters also minimize the need for repeated venipuncture [1,3].

Complications are categorized as early (within 30 days of placement) or delayed (after 30 days). Early complications include pneumothorax and catheter misplacement, while delayed complications include infection, thrombosis, and catheter fracture with migration [4]. Fracture and migration are uncommon and typically develop after prolonged use due to pinch-off issues, long-term wear, material fatigue or inadvertant pulling during removal. The early warning signs of catheter rupture include unexpected resistance or the catheter appearing shorter than usual after removal. This complication can result in the fragment lodging in the superior vena cava, right heart, or pulmonary arteries [5-7]. We report a successful percutaneous endovascular retrieval of a migrated catheter fragment from the right atrium using a snare.

## Case presentation

A 44-year-old female with a recent diagnosis of left buccal mucosa carcinoma was referred for endovascular removal of a fractured central venous catheter. The distal portion had detached and migrated during attempted removal from the right internal jugular vein. Patient did not develop any specific symptoms due to this embolisation. Given the risks of further migration and serious complications, urgent retrieval was indicated.

Procedure

**Figure 1:**
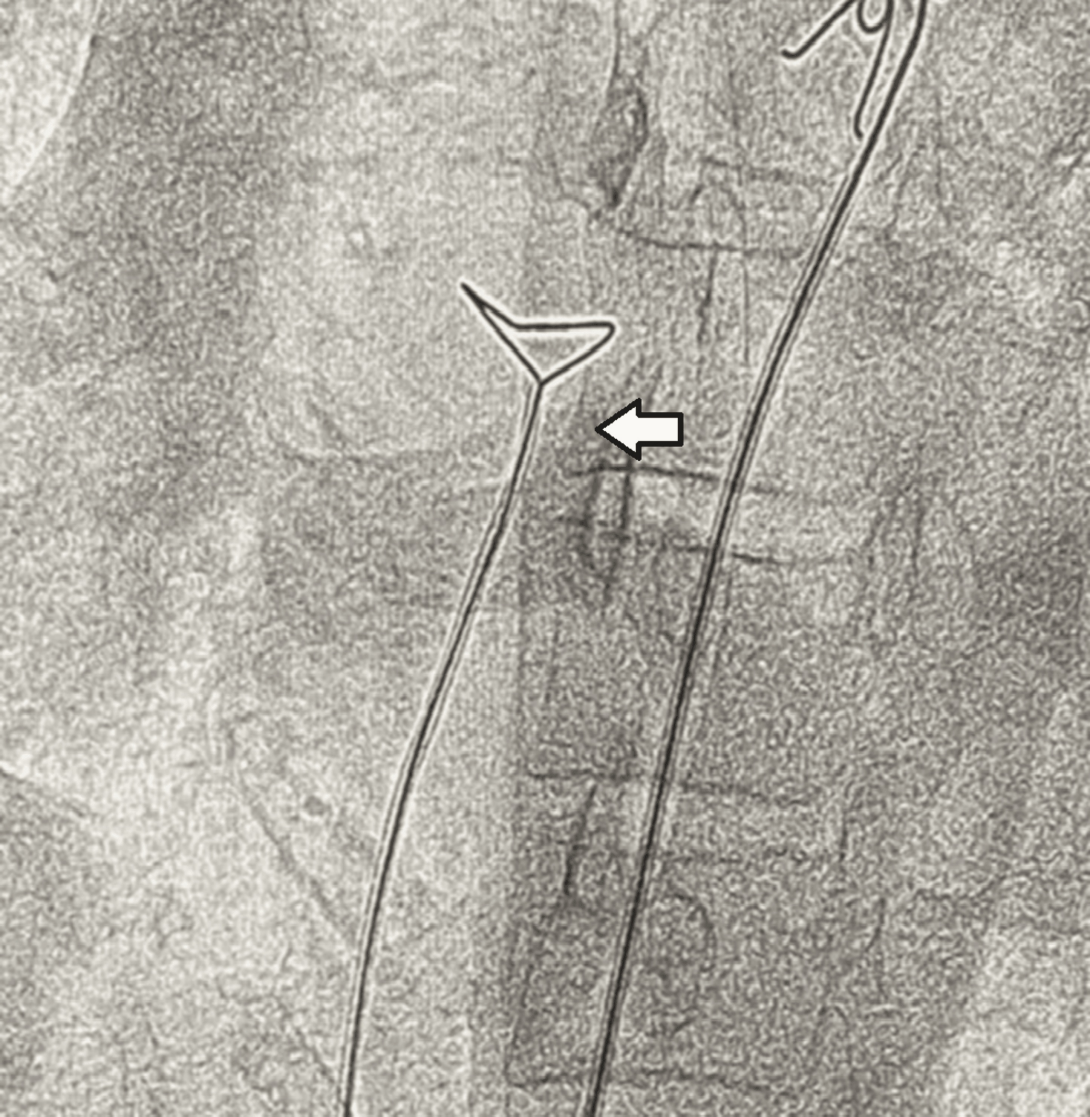
Snare catheter (arrow) through right femoral access. Biggest hardle was the visualisation of the embolized catheter.

Under local anesthesia, a 9 French femoral sheath was placed in the right femoral vein using the Seldinger technique. A hydrophilic guidewire was advanced into the inferior vena cava. A snare catheter was then navigated to the site of the embolized fragment (Figure 1). A significant technical challenge was the radiolucent nature of the catheter tip, requiring localization solely via high-resolution cine-imaging.

**Figure 2:**
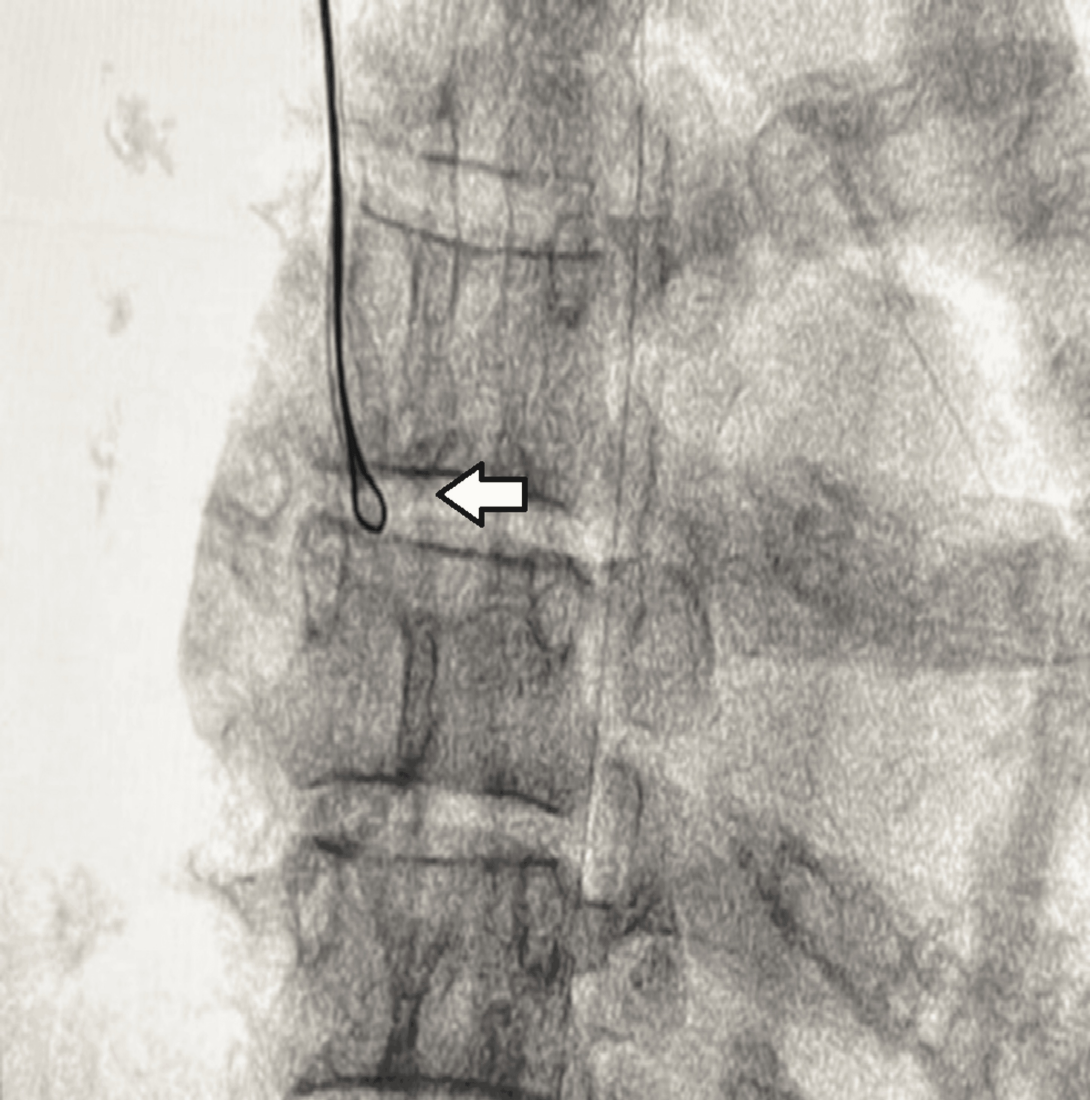
Snare catheter introduced through right internal jugular vein holding the embolized catheter (arrow)

**Figure 3:**
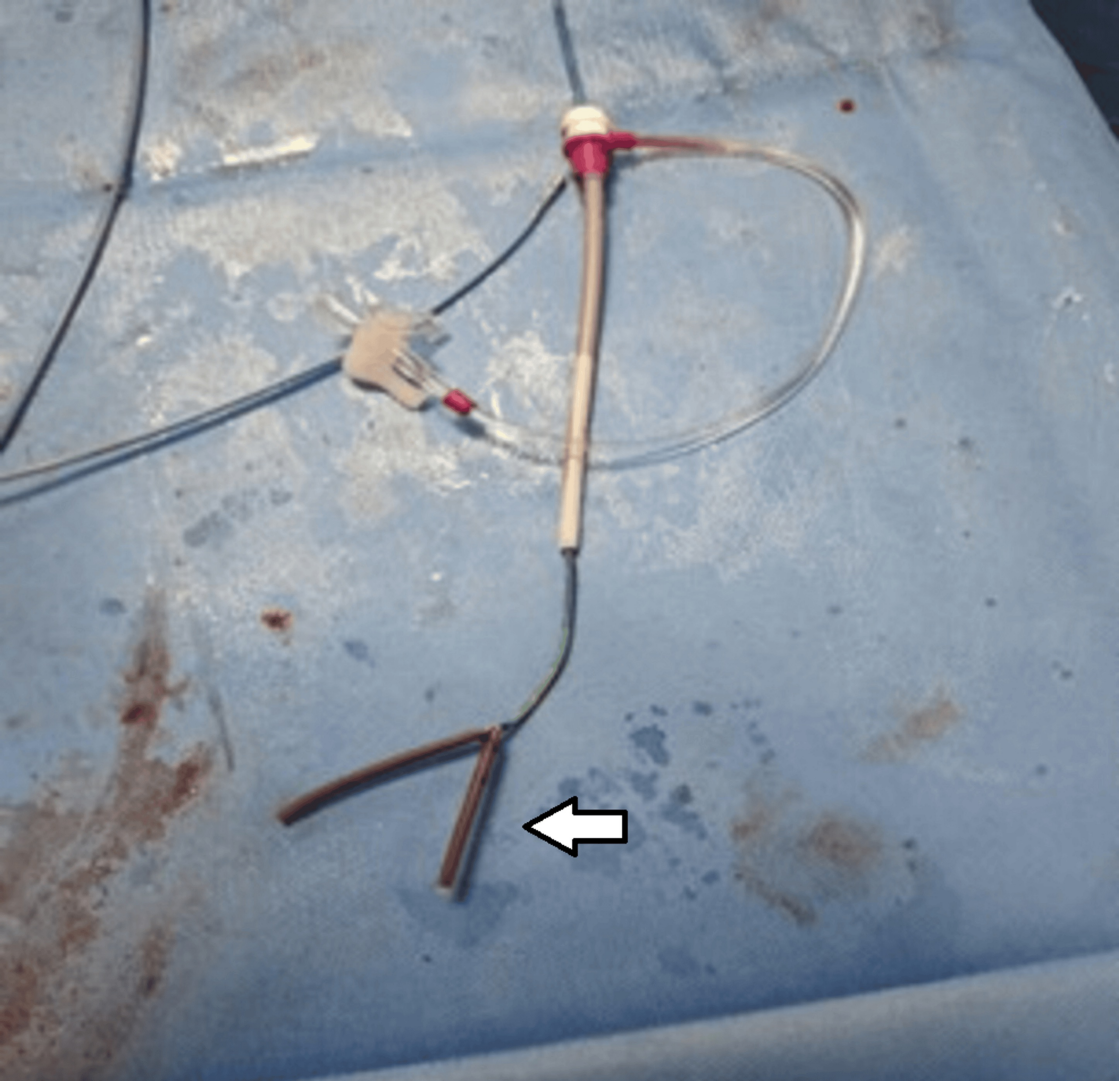
The retrieved broken catheter (white arrow) alongwith the attached snare catheter utilized during the retrieval procedure.

**Figure 4:**
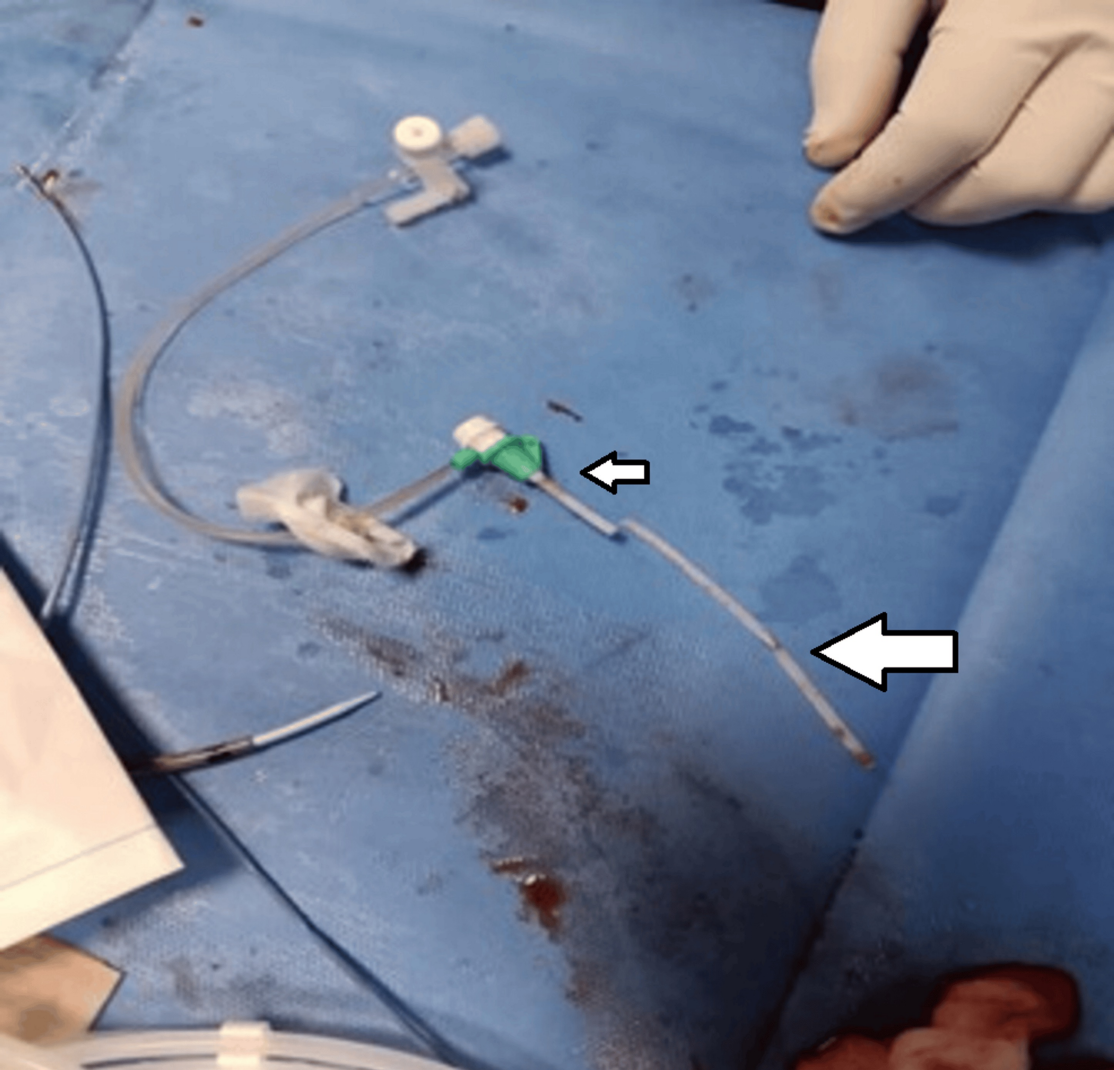
Recreation of the central venous catheter with the retrieved portion of distal embolized broken portion (larger arrow) alongside the base (smaller arrow)

After multiple attempts, one end of the fractured catheter was ensnared. However, due to suboptimal alignment, it could not be withdrawn into the inferior vena cava. During attempts to realign the system, the fragment dislodged from the snare and could not be recaptured. Consequently, the access site was changed to the right jugular vein (Figure 2). From this approach, the fragment was captured with fewer attempts. The entire system, including the jugular sheath, was then removed en bloc (Figures 3, 4).

After heparin reversal with protamine, hemostasis was achieved by manual compression. Total fluoroscopy time was 20 minutes. The patient was monitored continuously throughout the procedure, and a post-operative chest X-ray confirmed successful retrieval. Post-procedure echocardiography was also carried out to exclude any possible complications, such as pericardial effusion/cardiac tamponade. Patient went home next day without any subsequent events. 

## Discussion

Intracardiac foreign bodies (IFBs), often affecting the right heart, can be asymptomatic or lead to severe complications like infection, embolism, and arrhythmias [8]. The increasing use of long-term venous access devices has contributed to a higher incidence of IFBs [9].

Current management options for IFBs include open thoracotomy, endovascular therapy, and conservative management [10]. Surgery is more invasive and costly, whereas endovascular techniques boast success rates of 87-98% and are now first-line therapy, with surgery reserved for complex cases [11,12]. Conservative management remains controversial and is influenced by symptoms, foreign body size, and patient preference. Since 1964, various endovascular tools have been developed, including snares, balloon catheters, and forceps. The snare is the most common device. Balloon catheters are effective for cylindrical objects like stents, while baskets and forceps are useful in specific anatomical situations [12-15]. Fluoroscopy is the primary imaging modality for percutaneous retrieval, effectively visualizing most metallic foreign bodies [11]. Ultrasound can be utilized for radiolucent objects or those difficult to visualize due to cardiac motion [16,17].

Common complications of percutaneous retrieval include puncture site hematoma and transient arrhythmias from instrument contact with cardiac structures [18]. Overall, endovascular retrieval is considered safe, effective, and minimally invasive, expanding treatment options for fragile patients. While peripherally inserted central catheter (PICC) placement is generally safe, complications like dysfunction, infection, and occlusion can occur [14,15]. Catheter fragment embolization is a rare but dreaded complication that requires high clinical suspicion and immediate intervention [10]. An intravascular fragment lodged in the right heart can cause arrhythmia, hypotension, or valvular dysfunction. Distal embolization into a pulmonary artery carries a risk of thrombosis and infarction.

In our case, the catheter fractured near its base, embolizing as a single piece into the right heart. Fortunately, the patient remained hemodynamically stable. The fragment was successfully retrieved using a snare, and the patient was discharged without further complications.

## Conclusions

Fracture and embolization of central venous catheters, though rare, is a potentially life-threatening complication that requires prompt recognition and management. Endovascular retrieval is the preferred first-line approach due to its high success rate, safety, and minimal invasiveness. This case demonstrates the effective use of a percutaneous snare technique, underscoring the importance of procedural adaptability, such as changing vascular access, to overcome technical challenges. Radiolucent nature of the embolized foreign body may impose a major challenge for percutaneous retrieval, like in our case. The successful outcome reinforces that with timely intervention and expertise, nonsurgical retrieval of intracardiac catheter fragments can be performed safely and efficiently, even in technically challenging situations.
